# Contiguity and Structural Impacts of a Non-Myosin Protein within the Thick Filament Myosin Layers

**DOI:** 10.3390/biology10070613

**Published:** 2021-07-02

**Authors:** Lynda M. Menard, Neil B. Wood, Jim O. Vigoreaux

**Affiliations:** Department of Biology, University of Vermont, Burlington, VT 05405, USA; lynda.menard@uvm.edu (L.M.M.); neil.wood@uvm.edu (N.B.W.)

**Keywords:** insect flight muscle, thick filament, myosin, flightin, coiled-coil

## Abstract

**Simple Summary:**

Hexapods and crustaceans (Pancrustacea) represent nearly 80% of known living animals. Species within this clade exhibit exquisite muscle types propelling ingenious means of locomotion, likely contributing to their evolutionary success. Flightin, a myosin-binding protein, first identified in the flight muscle of Drosophila, is defined by WYR, a protein domain exclusive to Pancrustacea. In Drosophila, flightin imparts stiffness to the thick filament and is essential for their length determination and structural integrity. Here, we build on results from the three-dimensional reconstruction of the Lethocerus flight muscle thick filament to advance the hypothesis that flightin influences thick filament mechanics, and by extension muscle function, by acting as a cinch in the filament core.

**Abstract:**

Myosin dimers arranged in layers and interspersed with non-myosin densities have been described by cryo-EM 3D reconstruction of the thick filament in Lethocerus at 5.5 Å resolution. One of the non-myosin densities, denoted the ‘red density’, is hypothesized to be flightin, an LMM-binding protein essential to the structure and function of Drosophila indirect flight muscle (IFM). Here, we build upon the 3D reconstruction results specific to the red density and its engagement with the myosin coiled-coil rods that form the backbone of the thick filament. Each independent red density winds its way through the myosin dimers, such that it links four dimers in a layer and one dimer in a neighboring layer. This area in which three distinct interfaces within the myosin rod are contacted at once and the red density extends to the thick filament core is designated the “multiface”. Present within the multiface is a contact area inclusive of E1563 and R1568. Mutations in the corresponding Drosophila residues (E1554K and R1559H) are known to interfere with flightin accumulation and phosphorylation in Drosophila. We further examine the LMM area in direct apposition to the red density and identified potential binding residues spanning up to ten helical turns. We find that the red density is associated within an expanse of the myosin coiled-coil that is unwound by the third skip residue and the coiled-coil is re-oriented while in contact with the red density. These findings suggest a mechanism by which flightin induces ordered assembly of myosin dimers through its contacts with multiple myosin dimers and brings about reinforcement on the level of a single myosin dimer by stabilization of the myosin coiled-coil.

## 1. Introduction

Molecular-level muscle structure amongst both vertebrates and invertebrates employs many of the same building blocks and strategies for structural and mechanical attunement per organism. Striated muscle is known for its organized subcellular arrangement of protein filaments into regularly repeating structures known as sarcomeres. Attunement of largely conserved thick filaments, prominently composed of myosin dimers, is accommodated by changes within the myosin dimer sequence, myosin assembly, and protein addendums. The packing of myosin within the thick filament backbone is known to vary between vertebrates and invertebrates, and among invertebrates [1]. The significance of these differences and their implications in thick filament function and mechanobiology are not fully understood but are likely to underpin muscle-type functional differences and locomotory modalities. Such understanding can be realized in model systems for which information from molecular structures can be interpreted in light of mechanical, physiological, and organismal functional properties.

Cryo-EM studies by Hu et al. [2] have revealed the thick filaments of Lethocerus (Hemiptera) to be arranged in layers of associating myosin dimers through the engagement of their light meromyosin (LMM) regions, long C-terminal coiled-coiled rods. These layers of myosin dimers include additional proteins winding their way through the dimers of each layer and between layers. The pitch of the coiled-coil was found to be variable (60–126 Å) with areas of unwinding. Four unconnected non-myosin densities were found and assigned different colors (red, yellow, blue, and green) (Hu et al. Figure 5 and movie S3 [2]). The ratio of each of the densities to myosin was found to be 1:1 with their combined volume amounting to 20 kDa of polypeptide, resulting in an expectation that each density represents an ordered protein segment whose less ordered regions are not visible. The red and yellow densities both connect to adjacent rods and contact the paramyosin core. They were characterized distinctly as the red density passes through from the surface to the center of the thick filament and the yellow density appears to ‘stitch’ together multiple layers. The former was hypothesized to be flightin [3] and the later to be myofilin [4].

The position of the red density is the primary reason for flightin’s attribution. Flightin is known to be a component of the Drosophila thick filament [5] and to bind within a 600 amino acid segment of the LMM in vitro [6]. The mutation E1554K in Drosophila myosin prevents flightin accumulation in vivo [7] and binding in vitro [6]. The red density is found on the outside of the filament, consistent with flightin antibody labelling in Lethocerus [8], and in close proximity with the rod at the corresponding E1554 residue in Lethocerus (E1563). The flightin to myosin stoichiometry was calculated to be approximately 1:1 to 1:2 [6], in alignment with the 1:1.4 to 1:2 ratio suggested by Hu et al. [2].

## 2. Materials, Methods and Results

We set out to determine the specific amino acid regions and the pattern of red density contacts with the LMM as it winds its way through the myosin dimers of the thick filament. The 3D model of the thick filament, inclusive of non-myosin densities, is provided in movie S3: a video fly-through which follows the complete path of a myosin dimer in the M-ward direction, as viewed from the globular head to the end of the coiled-coil rod [2]. The key to the video provided in Hu et al. Figure S8 [2] was used along with a manual matching procedure, using ApowerEdit [9], to properly orient the LMM sequence encompassed in each frame. The video was sorted into three 435 Å segments and one 292 Å segment, and the primary region of interest (G1528-A1628) was determined within the boundaries of its 435 Å segment. The frames for this section had a representative dimer isolated from the rest of the image using GIMP [10]. The resultant images were evaluated in ImageJ [11,12] (see Supplementary Materials).

**Figure 1:**
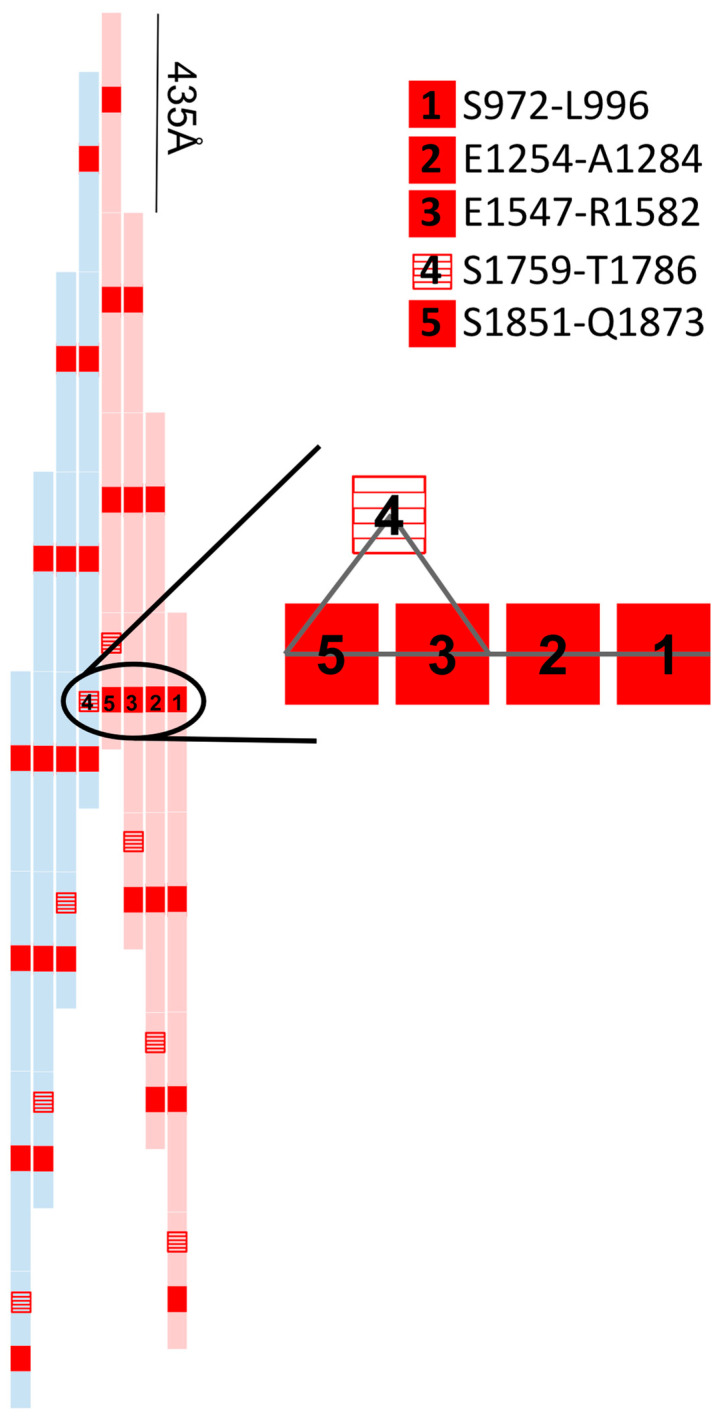
Each myosin contains a 1600 Å rod region that overlaps with each other such that 3–4 dimers (rods) are aligned at any one time at a stagger of 435 Å. The red rectangles represent the red density contact sites along the length of four dimers. The numbers 1–5 show the LMM interfaces for a single red density, linking each rod to the other rods in its layer and once to a neighboring layer (4, red stripes). The area of position 3–4–5 is the multiface.

**Figure 2:**
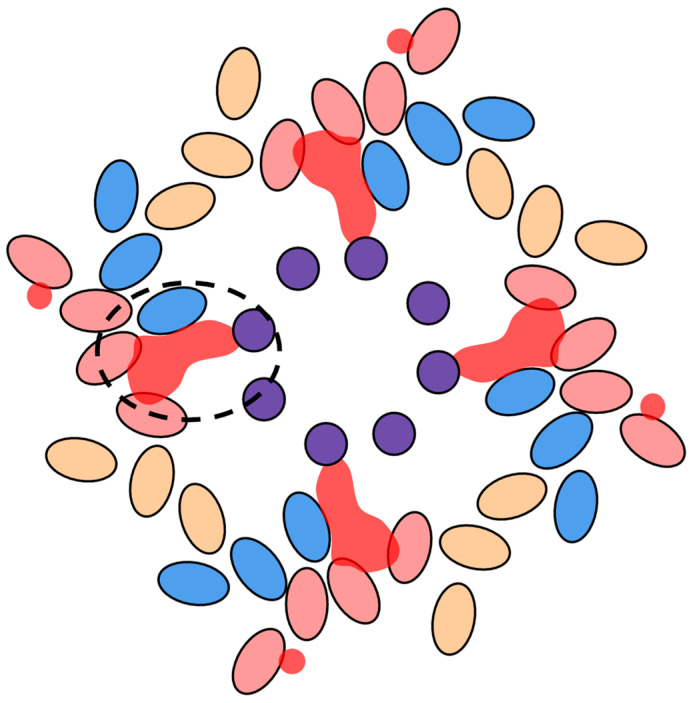
Graphical view down the filament axis (redrawn from a frame of Hu et al., movie S3 [2]). Ovals represent myosin dimers with the color (pink, yellow, blue) representing individual layers. The red density is shown in a translucent red and is circled at the multiface (dashed oval). Here, the red density contacts three LMM interfaces simultaneously with two dimers belonging to the ‘pink’ layer and one dimer belonging to the ‘blue’ layer. Contact with paramyosin (purple circles) is made at the center of the thick filament.

The winding path that the red density takes along the length of the myosin rod brings it in close contact with five different sections of the LMM. Among these are four sections within the same layer (S972-L996; E1254-A1284; E1547-R1582, and S1851-Q1873) and one section in a neighboring layer (S1759-T1786) (Figure 1). A single red density contacts each of these regions once along five different myosin dimers, alluding to a possible role in tying or clasping them together. We denote the last three contact areas (E1547-R1582; S1759-T1786; S1851-Q1873) the “multiface”, because a single red density is simultaneously contacting three distinct myosin dimers (Figure 2). The multiface is of further interest as this is where the red density links layers and reaches the thick filament core to contact paramyosin.

The linking of four dimers within a layer to each other and to one dimer in a neighboring layer may represent a mechanism whereupon flightin directs and secures ordered assembly of myosin into the thick filament. Skinned IFM fibers from the mutant *Df(3L)fln^1^*, which results in 20% less flightin, exhibit a loss of thick filaments from the myofibril periphery as if these myosin molecules were not firmly secured in the outwardly developing myofibril [13]. In the flightin null mutant *fln^0^*, there are decreased thick filaments across the myofibril diameter and sarcomeres and thick filaments are 25% longer and more variable in pupae with breakdown occurring shortly after eclosion [5]. Such change in the arrangement of thick filaments within the myofibril coupled with instability throughout the *fln^0^* muscle system speaks to flightin’s role as conducive and secure to higher order structure myosin assemblies.

Given the prevalence of coiled-coils in proteins with mechanical roles [14,15] and the importance of the myosin coiled-coil in assembly of the thick filament [16], we asked whether the red density was associated with any changes in the winding (pitch) of coiled-coil structure. The structure of the coiled-coil is guided by a heptad repeat, residues in the pattern of HPPHCPC in which hydrophobic (H), polar (P) and charged (C) residues dictate the left-handed supercoiling of the right-handed helices [17]; however, deviations from this pattern are common and contribute to variation in the coiled-coil pitch [18]. Rotational angle change relative to the major axis of the dimer was measured M-ward along the length of the LMM, from G1528 to A1628, for several matching layers and averaged. To do this, an isolated dimer was fit to an ellipse in ImageJ and the angle change between frames was recorded and graphed against the associated amino acid range (see Supplementary Materials).

**Figure 3:**
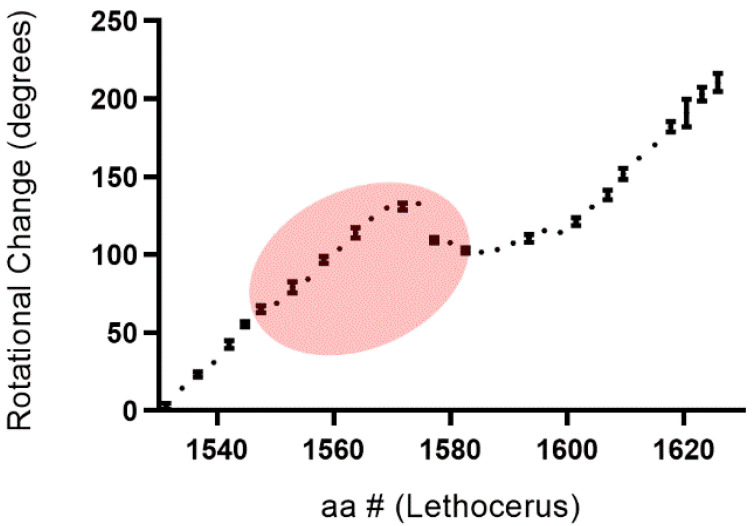
M-ward helical rotation of the coiled-coil from G1528-A1628 where the change in angle represents rotational change between frames. Each point is averaged across three dimers within the same layer ± SD. The points within red shading is the area over which the red density is contacting the myosin dimer.

We find negative slope and stasis of rotation between S1574 and R1582, representing a change in apparent direction of the dimer turn to be slightly right-handed (Figure 3). Generally, there is an M-ward left-handed turning of the dimer. This indicates change in the winding of the coiled coil: a local relaxation of pitch. This area is especially interesting as the red density making contact with the dimer also contacts paramyosin over a short span from E1572 to Q1575. Once the red density disappears, around R1582, the slope recovers its typical rotation. This precedes the third skip residue (E1590). When rotation is mapped along the entire myosin rod, similar shifts are evident only in locations associated with skip 1 (T1196) and, possibly, skip 4 (G1815), centering around T1196 and Q1802 (not shown).

The association of the red density with a change in pitch proximal to E1590 could help stabilize an otherwise unstable area. Few crystal structures of portions of the LMM exist though it has been shown that the skip residues are responsible for disruption in the coiled-coil that extends beyond a heptad both N- and C-terminally [16,19] in the absence of non-myosin thick filament proteins. If the red density stabilizes the area N-terminal to the third skip residue, it can reinforce the coiled-coil by preventing the disturbance from radiating further. Such stabilization of the coiled-coil may be taking place in other areas of the LMM in connection to the red density, or other non-myosin densities, as the LMM is known to harbor additional deviations (stammers, stutters) from the heptad ideal for coiled-coil formation [17]. Securing areas of heptad disruption would increase the overall coiled-coil integrity of the LMM and resilience in the context of contractile forces.

**Figure 4:**
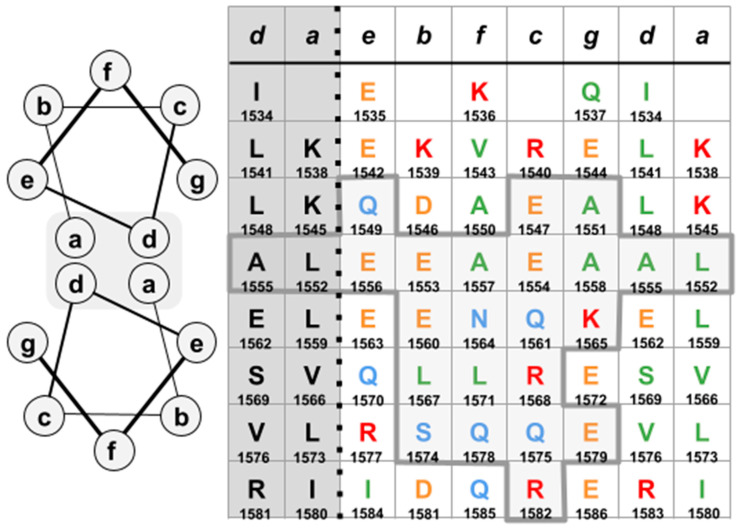
(**left**) Heptad position designation in a classical coiled-coil. (**right**) Interface over which red density was proximal from perspective of heptad positions ((**a**–**g**), top row). Note ’d’ and ’a’ positions are repeated (dark shade). Thick grey lines and light shading designate the amino acids in contact with the red density.

The LMM region in the vicinity of E1563 (E1554 in Drosophila), spanning from residues E1547 to R1582, was further examined to identify potential residues in direct juxtaposition to the red density. The specific interface of the LMM to the red density was defined based on the angular relationship of the red density to each monomer in the LMM dimer. Notation is not taken beyond a 5-pixel distance (3.2 Å); this relationship is estimated using Hu et al. Figure 4A [2] (see Supplementary Materials).

The residues identified are shown in Figure 4. Other residues in this area may be important for the orientation of the LMM relative to the red density as this region is part of the multiface and is stabilized by other dimers. The contact region borders E1563 (E1554 in Drosophila) and contains R1568 (R1559 in Drosophila). The inclusion of these two residues within the interface, in contact with the bulk of the red density, provides an explanation for the depletion of flightin in Mhc^13^ and Mhc^6^, two Drosophila strains that carry point mutations E1554K and R1559H, respectively [7]. These mutations significantly diminish power output while differing in their effects on fiber passive and dynamic viscoelastic properties [20]. The revelation of this interface allows further exploration on the nature of the flightin–myosin interaction and consequences on thick filament structure, fiber mechanics, and muscle function.

The estimated mass of the red density is less than the mass of flightin [3]. Mutant Drosophila flightin lacking the N-terminal 62 amino acid region [21,22] or the C-terminal 44 amino acid region [23] are incorporated into the fiber, indicating that the region between amino acids 63 and 138, encompassing the conserved WYR domain [24], harbors an essential myosin binding site. We hypothesize that the red density is mostly or exclusively WYR, further supported by the predicted unstructured nature of the larger N-terminal region [25]. The myosin sequence encompassing the red density interface is well conserved between vertebrates and invertebrates. Comparison of the Drosophila MHC rod sequence to its human cardiac counterpart reveals 56% identity (74% positives) while the interface in the area of flightin binding between I1534-E1586 shares 68% identity (85% positives). Vertebrate proteins that influence thick filament stability and alignment, including M-protein, myomesin and Myosin Binding Protein-C (MyBP-C), have been shown to bind to this region [26,27,28]. A shared myosin coiled-coil binding region raises the prospect of a conserved binding mechanism for these divergent proteins. Furthermore, studies with exogenously expressed cardiac MyBP-C in wild-type and *fln^0^* flies suggest that flightin and cMyBP-C have partially convergent functions, both in contributing to the mechanical properties of the thick filament (flexural rigidity) and in assembly (thick filament and sarcomere length) [21,29,30].

The findings from this study provide insight into the mechanism by which flightin provides stability and rigidity to the thick filament as arising from securing of the LMM coiled-coil and enforcement of dimer-dimer contacts within the thick filament. We propose that flightin behaves as a ‘cinch’ to stabilize the LMM structure and segment the coiled-coil thereby influencing the thick filament’s capacity for mechanical relay and stretch activation. The highly conserved region of binding on the LMM may further allude to a shared strategy between invertebrate and vertebrate striated muscle for tuning thick filament properties. The information presented can inform molecular dynamics and structural studies to shed light on a possible conserved mode of molecular interaction between the myosin coiled-coil and its binding partners.

## 3. Conclusions

In this study, we built upon the high resolution cryo-EM structure of the Lethocerus flight muscle thick filament obtained by Hu et al. [2] to further characterize the area of myosin contact associated with the red density, attributed to flightin. In doing so we unveil a possible mechanism by which flightin modulates the thick filament through the interaction of its conserved WYR domain with myosin coiled-coils. We bring emphasis to the red density participating in an M-ward order of five LMM contact sites, entering the thick filament core at a multiface where it simultaneously contacts three dimers, between two layers. We examine the coiled-coil in the context of the red density to find that the area of the LMM contacting the red density between E1547 and R1582 is associated with a local unwinding of coiled-coil pitch, in which the coiled-coil recovers from the N-terminal disruption extending from the 3rd skip residue, while in contact with the red density. This area is known to contain residues that, when mutated in Drosophila, inhibit flightin accumulation in the flight muscle and lead to myosin degradation and sarcomere breakdown. Taken alongside prior studies demonstrating the essential role of flightin in dictation of thick filament mechanical properties, dimensions and stability within the sarcomere, these findings allude to a mechanism for flightin’s stabilizing effects arising from securing of the LMM coiled-coil and enforcement of dimer-dimer contacts within the thick filament. In light of recent in vitro binding studies demonstrating that WYR alters the structural properties of the LMM (Menard et al., Biology, in press), we propose the WYR: LMM interaction sites behave as a cinch to stabilize the LMM structure and to segment the coiled-coil, thereby influencing the thick filament’s capacity for mechanical relay and stretch activation. The LMM interface further articulated between E1547 and R1582 can be used to inform future research into WYR’s role in flightin function and structural–mechanical adaptations in muscle.

## Data Availability

The data presented in this study are available in the article entitled “*Contiguity and structural impacts of a non-myosin protein within the thick filament myosin layers*”, the accompanying supplement, and here https://scholarworks.uvm.edu/graddis/1341/.

## Supplementary Materials

The following are available online at https://www.mdpi.com/article/10.3390/biology10070613/s1, Figure S1: The heptad is mapped as two right-handed alpha helices participating in a left-handed coiled coil. Table S1: Angstrom values used for calculations in the analysis of movie of S3 [1]. Table S2: Comparison of time span and duration methods. Table S3: Calculating Values for Major Axis Line.
