# Supplementary material for: Contiguity and Structural Impacts of a Non-Myosin Protein within the Thick Filament Myosin Layers
Source: Biology (Basel). 2021 Jul 2;10(7):613. doi: 10.3390/biology10070613 (PMC8301149; doi:10.3390/biology10070613)
Supplement: Supplementary file 1 [file biology-10-00613-s001.zip › biology-1260873-SI.pdf]

Supplementary Material to accompany the article "Contiguity and structural impacts of a non-myosin protein within the thick filament myosin layers"

Lynda M. Menard, Neil B. Wood, and Jim O. Vigoreaux

Overview

This supplement contains further processing information of Supplementary movie 3 [1], "Cross-sectional thick filament fly-through", an M-line directed fly-through of the myosin dimers participating in the 12 curved ribbon-like layers of the thick filament. The video follows a single dimer as it winds its way through the thick filament to its core. Along the way, it makes contact with multiple other myosin dimers and non-myosin densities. This single dimer is termed 'cyan' for its coloration in the video and is referred to as it was used for orientation to the LMM sequence. It is a member of the 'blue' layer. Initial measurements were fit to the Drosophila myosin sequence, as was used in Hu et al., 2016 [1] and were later accommodated to fit the Lethocerus myosin sequence. The heptad designations for the LMM residues were limited to G1528-A1628, based on Taylor et al 2015 [2].

We address three main processes, organized as follows:

1. Identifying start/end points for the selected dimer
    a. Characterizing the structure of the video
    b. Selecting the region of interest
2. Mapping the rotation of the selected dimer
    a. Isolating the dimer fit to an ellipse
    b. Attaining coiled-coil rotation over set aa range
3. Attaining angle of interface of dimer to the red density

Identifying start/end points for the selected dimer

Characterizing the structure of the video

Values important to calculations over the duration of the movie are shown in Table S1. The movie S3 is described as going through 1700 Å which encompasses an entire myosin dimer. The entire video is just over 28 seconds long with 15 fps (~420 frames).

Table S1: Angstrom values used for calculations in the analysis of movie S3 [1].

1700 Å	Myosin distance encompassed by Movie S3
1598 Å	Contour length of myosin rod and tether
435 Å	Stagger between rods in a ribbon layer
4 Å	Distance between 2 frames
1.485 Å	Rise per residue relative to coiled-coil axis

While the video itself is ~420 frames, 1.3 to 27.9 sec (a span of 26.6 sec) represents the extent of the cyan dimer rod region. The full length of the rod, including tether is reported as 1598 Å. At 15 fps, 26.6 sec

calculates to 399 frames (26.6 sec*15 fps). With each frame being 4 Å apart, the expected number of frames is 399.5 (1598 Å /4 Å) which supports the previous calculation.

There are crowns every 145 Å with a 435 Å axial repeat for crowns along the length of the thick filament for which dimers in any individual layer laterally associate. When following the cyan dimer, each blue crown is 435 Å from either neighboring blue crown. After the cyan crown, the next blue crown is 435 Å from that start point and so forth. As there is a 1.485 Å rise per residue, the number of amino acids in 435 Å is approximately 293 (435 Å /1.485 Å = 292.93). This corresponds to the number of amino acids along a dimer between crowns originating from the same layer.

Figure S8 [1] is a 'key' to the S3 movie and designates 1.3, 8.6, 15.7, and 22.9 seconds as crowns spaced 435 Å apart along the cyan myosin dimer tracked in Movie S3. The time between these sections is not consistent but averages to 7.2 seconds indicating that 108 (7.2*15) frames as representative of a 435 Å region. As there are 7.2 seconds between crowns and 15 fps, there are 108 frames per 435 Å. This is in close alignment with each frame being 4 Å along (435 Å /4 Å = 108.75).

Table S2 (gray columns) shows the span time and duration key provided in Figure S8 [1]. Using 1.3 sec as the starting point, we used the video editing software, ApowersoftEdit [3], to assign the span and duration more precisely through closest matching of the myosin head densities at crown positions, every 435 Å using 1.3 sec as the starting point (Table S2, right-most columns). These were considered our checkpoints. The 7.25 second durations fits better with the calculated number of frames per 435 Å (108.75 frames).

Note, in ApowersoftEdit, seconds are separated into 1/20s with a notation that completes at "0.2". For example, 4:30 seconds, which equates to 4.5 seconds in decimal notation, is expressed as 4.1 in ApowersoftEdit.

Table S2. Comparison of time span and duration methods

	S8 key without matching		Adjusted key with matching	
AA seg Dros	Span (s)	Duration (s)	Span (s)	Duration (s)
843-1136	1.3-8.6	7.3	1.3-8.55	7.25
1136-1428	8.6-15.7	7.1	8.55-15.8	7.25
1428-1721	15.7-22.9	7.2	15.8-23.05	7.25
1721-1919	22.9-27.76	4.86	23.05-27.91	4.86

The detectable rod length and tether is 1077 residues (R843-F1919). This is 1598 Å with ~293 aa per 435 Å segment. 1598 Å is 3.67 times 435 Å so the extra portion corresponds to (0.67*435 Å) 292 Å, or ~196 aa.

Calculations are as follows:

Distance after last same-layer crown until dimer disappears: 0.67* 435 Å = 291.45 Å
Frames covering last ~292 Å: 291.45 Å/4 Å = 72.8625 frames
Expected duration from last crown to last frame: 72.86 frames/15 fps = 4.86 sec
Expected end point after last participating crown following Figure S8 key [1]:
22.9 sec+4.86 sec =27.76 sec
Expected end point for adjusted crown positions by timepoint matching: 23.05 sec+4.86 sec = 27.91 sec

The expected end point with adjusted crown positions corresponds exactly with the last frame in which the cyan dimer is present. This supports the accuracy of the modified time points/frames associated with the residue ranges.

Selecting the region of interest

The entirety of the rod and tether is 1598 Å in contour length and identified, after fitting with the Drosophila sequence, to be 1077 aa in length from R843-F1919 with a rise-per-residue of 1.485 Å. At the time of the Hu et al. 2016 publication [1], the Drosophila sequence was used to fit the model as the Lethocerus sequence had not yet been published. The Drosophila region of interest selected for our analysis (R1520-D1620) falls within the third 435 Å region, with the rod being segmented into three 435 Å regions plus an additional 292 Å (Table S2).

Within the selected region exists two specific amino acids that are highly conserved and suspected to be involved in interaction between flightin and the LMM: Drosophila myosin residues E1554 and E1580 [4]. This area also contains the third skip residue (E1581). In Lethocerus, E1563 and E1589 correspond to Drosophila myosin residues E1554 and E1580. The skip residue is E1590 for Lethocerus. The total range from Drosophila R843-F1919 corresponds to Lethocerus N852-F1928. The R1520-D1620 Drosophila region corresponds to Lethocerus R1529-D1629.

Frames were separated using VLC. The region of interest aimed for, Lethocerus amino acids N1530-I1630, was manually separated out and designated frame #1-37. This ended up specifically being positions 1528.6 to 1628.5 and annotated as G1528-A1628.

Each frame was considered to be representative of a 4 Å region, which has 2.7 amino acid increments. The positioning of G1528-A1628 was calculated based on distance from the closest crown checkpoint.

Mapping the rotation of the selected dimer

Isolation of the cyan dimer is confounded by the black line overlay designed to show the path of movement through its layer over time. The diagonally opposite dimer to the cyan dimer was selected as representative. This exactly opposite dimer moves in the exact same pattern as the cyan dimer.

After isolation of the 37 frames, the representative dimer was separated from the rest of the image using GIMP [5] and saved as .png and .jpg files. The following progression was done to convert the selected dimer to neonpink to distinguish the selected dimer from the other dimers in the ribbon in each frame:

GIMP Protocol

1. (Colors -> Adjust Brightness and Contrast) Added 10% contrast
2. Selected dimer of interest
3. (Colors -> Adjust Brightness and Contrast) Added 10% contrast
4. (Colors -> Adjust Color Balance) Set to Grey (Midtones, maxed)
5. (Colors -> Adjust Color Balance) Highlights, maxed to Red
6. (Colors -> Colorize) Maxed Hue and Saturation

In the above progression, the contrast and brightness of the entire image was enhanced. A small area, inclusive only of the dimer of interest and background, was selected and further brightened and contrasted. Color balance was then adjusted to starken the color contrast of the dimer from the rest of the image. Frames were saved as PNG files for analysis in ImageJ [6].

Isolating the dimer to fit an ellipse

ImageJ Protocol
In order to attain an axis for each frame over which position and rotational change can be tracked, we fitted each dimer to an ellipse that encompassed both myosin monomers.

1. (Image>Adjust->Color Threshold) Threshold Color set to point just before selection circle is not evident around dimer of interest: Hue 0, 36; Sat 5, 255; Brightness 130, 255
2. (Analyze->Set Measurements) 'Area', 'centroid' and 'fit ellipse' were checked.
3. (Analyze->Analyze Particles) Set min pixel size to 35 to reduce off-target measures
4. Set measurements to fit ellipse and centroid. This automatically selected the two globular units as a single ellipse. Measurements of the major axis angle and the length of the major (long) and minor (short) axes of the ellipse were provided by the program under these settings.
    - The "Draw Ellipse" macro was used to visualize the measured ellipse. (Edit->Selection->Fit Ellipse)

Attaining coiled-coil rotation over amino acid range

We used the measurements of the ellipse to track movement of the dimer over a series of frames. ImageJ fitting provided X,Y coordinates of center of ellipse, length of major and minor axis line, and angle of major axis.

The angle of the major axis represents the major axis line's angle relative to the horizontal of the image out of 180 degrees. To check, the angle function was used while going over the line used to form Ellipse using the "Draw Ellipse" macro. These values were collected from each frame and the angle change between frames was recorded and graphed relative to frame 1 (set to zero degrees) against the associated amino acid range with each frame progressing 2.7 amino acids. This was then graphed in sequence to visualize the rotational shift. We did this for the 100 amino acid range of multiple dimers within the blue layer and dimers within the yellow and pink layers (n=6) to confirm conservation among various dimers within the same region (not shown).

When mapping the entire dimer over the full range for the visible dimer there will be some discrepancy due to the increased span over which drift will occur. Drift is resultant from the actual frame span being very slightly off from 4 angstroms and 2.7 aa per frame being a rounded value. Small imperfections are magnified over long spans that are not evident within a 100 aa range. The discrepancy is less than 1 frame. Frames 60-411 have been evaluated both with and without a forced fit to the check points. There is no substantial loss of information and the regions over which there are rotational shifts are consistent. We chose a forced fit to the check points as this equalizes the drift between segments and decreases the overall drift.

Linear regression analysis was done using Graphpad PRISM. R-square values were very similar regardless of whether residue numbers are fixed to checkpoint-values (0.9977 vs 0.9976) suggesting that the methods are comparable for making observations over larger amino acid regions. Checkpoints are useful for aligning heptad positions (next section) as they provide better accuracy by ablating drift.

Attaining angle of interface of dimer to the red density

ImageJ Protocol

1. We calculated the X,Y coordinates for the start and end points of the major axis, manually inserted those values into a new Draw Line macro, and ran the macro on the image.

Table S3. Calculating Values for Major Axis Line	
Start X	Xg-(COS(Ag*PI()/180)*(Lma/2))
Start Y	Yg+(SIN(Ag*PI()/180)*(Lma/2))
End X	Xg+(COS(Ag*PI()/180)*(Lma/2))
End Y	Yg-(SIN(Ag*PI()/180)*(Lma/2))
Center point (Xg,Yg), Angle (Ag), and Length (Lma) are given:	
Xg	Given X
Yg	Given Y
Ag	Given Angle (Major)
Length for Major	Lma

2. Used the reflex angle feature to trace over the drawn line representing the major axis such that the angle parallel to the line is 180° to 360°.
3. Measured the angle range over which the dimer is facing the density of interest.
   - Subtracted 360° from any value that passes the 360° point parallel to the major axis to get the 'actual' value.
4. Compared the angle ranges to the heptad positions
   - The coiled coil is left-handed and composed of two right-handed helices. A predominant left handed rotation is seen when viewing the frames in video S3 [1]. The arrangement of the two helices in accordance to heptad positions is show in figure S1.

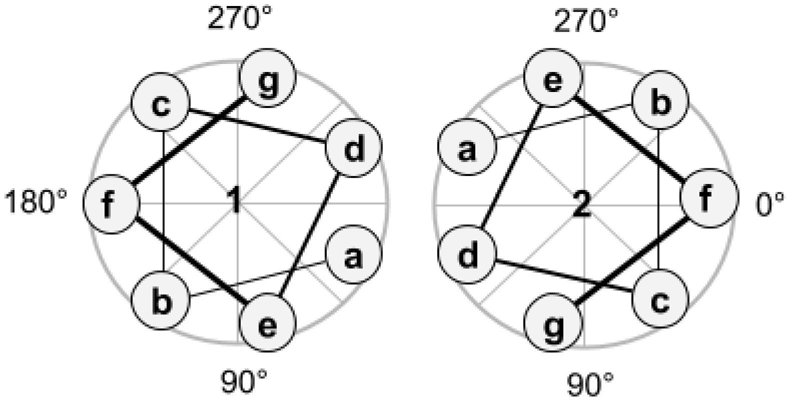

Figure S1. The heptad is mapped as two right-handed alpha helices participating in a left-handed coiled coil. Because angle measurements are not the same for each helix along the major axis, the helices are given arbitrary "1" and "2" designations.

Within the 37 frames of the selected region, the red density appears from frame 7 to 19. The dimers turn counter-clockwise in this M-ward view, therefore the alpha helices are turning clockwise [7]. The heptad positions are viewed to be in the format shown in Figure S1.

To determine the amino acids that are in close contact with the red density, the dimer was identified as having alpha helices "1" and "2" since at different points only one of the two might be close to the red density or the two helices may be close to the red density at different angles. Helix "1" was designated to be the left helix (Fig. S1). For the duration of the 37 frames, the helices do not switch relative position (ex. helix 1 is always left-most).

Although the rods rotate over time, the major line of the ellipse formed between the two densities was considered to be zero to 180° as the horizontal major axis in Fig. S1. If, for instance, the red density is closest to Helix 1 (the left helix in Fig S1), between the angles of 230°-300° then positions 'c' and 'g' are noted as being in closest proximity and it depended on the range encompassed by the frame whether 'c' or 'g' is determined to be the closest contact.

Notation is not taken past a 5 pixel distance (~3.2 Å); this relationship is estimated using Fig. 4A in [1].

Cumulative mapping of the myosin dimer to the red density across frames 7-19 allowed positions to be identified for amino acids involved in the interfacing between the two. This combined with dimer rotation measurements allowed correlation of pitch change along the region of interest.

The information presented here and other relevant information can be found in https://scholarworks.uvm.edu/graddis/1341/.

1. Hu, Z., et al., *Structure of myosin filaments from relaxed Lethocerus flight muscle by cryo-EM at 6 A resolution.* Sci Adv, 2016. **2**(9): p. e1600058.
2. Taylor, K.C., et al., *Skip residues modulate the structural properties of the myosin rod and guide thick filament assembly.* Proc Natl Acad Sci U S A, 2015. **112**(29): p. E3806-15.
3. Ltd A. ApowersoftEdit. https://www.apowersoft.com; 2017.
4. Kronert, W.A., et al., *Defects in the Drosophila myosin rod permit sarcomere assembly but cause flight muscle degeneration.* J Mol Biol, 1995. **249**(1): p. 111-25.
5. Team TGD. GIMP. https://www.gimp.org; 2017.
6. Schneider CA, Rasband WS, Eliceiri KW. NIH Image to ImageJ: 25 years of image analysis. Nature methods. 2012;9:671-5.
7. Peckham, M. and P.J. Knight, *When a predicted coiled coil is really a single α-helix, in myosins and other proteins.* Soft Matter, 2009. **5**(13): p. 2493-2503.
